# Automated Left Ventricle Ischemic Scar Detection in CT Using Deep Neural Networks

**DOI:** 10.3389/fcvm.2021.655252

**Published:** 2021-07-02

**Authors:** Hugh O'Brien, John Whitaker, Baldeep Singh Sidhu, Justin Gould, Tanja Kurzendorfer, Mark D. O'Neill, Ronak Rajani, Karine Grigoryan, Christopher Aldo Rinaldi, Jonathan Taylor, Kawal Rhode, Peter Mountney, Steven Niederer

**Affiliations:** ^1^School of Biomedical Engineering and Imaging Sciences, King's College London, London, United Kingdom; ^2^Department of Cardiology, Guy's and St Thomas NHS Foundation Trust, London, United Kingdom; ^3^Siemens Healthineers, Forchheim, Germany; ^4^3DLab, Sheffield Teaching Hospitals NHS Foundation Trust, Sheffield, United Kingdom; ^5^Siemens Healthineers, Medical Imaging Technologies, Princeton, NJ, United States

**Keywords:** computed tomography angiography, deep learning, convolutional neural network, fibrosis, left ventricle, automated classification

## Abstract

**Objectives:** The aim of this study is to develop a scar detection method for routine computed tomography angiography (CTA) imaging using deep convolutional neural networks (CNN), which relies solely on anatomical information as input and is compatible with existing clinical workflows.

**Background:** Identifying cardiac patients with scar tissue is important for assisting diagnosis and guiding interventions. Late gadolinium enhancement (LGE) magnetic resonance imaging (MRI) is the gold standard for scar imaging; however, there are common instances where it is contraindicated. CTA is an alternative imaging modality that has fewer contraindications and is faster than Cardiovascular magnetic resonance imaging but is unable to reliably image scar.

**Methods:** A dataset of LGE MRI (200 patients, 83 with scar) was used to train and validate a CNN to detect ischemic scar slices using segmentation masks as input to the network. MRIs were segmented to produce 3D left ventricle meshes, which were sampled at points along the short axis to extract anatomical masks, with scar labels from LGE as ground truth. The trained CNN was tested with an independent CTA dataset (25 patients, with ground truth established with paired LGE MRI). Automated segmentation was performed to provide the same input format of anatomical masks for the network. The CNN was compared against manual reading of the CTA dataset by 3 experts.

**Results:** Note that 84.7% cross-validated accuracy (AUC: 0.896) for detecting scar slices in the left ventricle on the MRI data was achieved. The trained network was tested against the CTA-derived data, with no further training, where it achieved an 88.3% accuracy (AUC: 0.901). The automated pipeline outperformed the manual reading by clinicians.

**Conclusion:** Automatic ischemic scar detection can be performed from a routine cardiac CTA, without any scar-specific imaging or contrast agents. This requires only a single acquisition in the cardiac cycle. In a clinical setting, with near zero additional cost, scar presence could be detected to triage images, reduce reading times, and guide clinical decision-making.

## 1. Introduction

Imaging cardiac scar is clinically indicated to assist in diagnosis, patient selection, risk assessment, and guiding heart failure therapies (1). Identifying whether a patient has scar present is important in procedure planning such as pacemaker implantation (2). Location of scar can impact treatment outcomes, with the influence of basal scar differing from scar at the apex (3, 4).

Late gadolinium-enhanced (LGE) magnetic resonance imaging (MRI) is the clinical gold standard in cardiac scar imaging (1). However, there are many patients where MRI with LGE is contraindicated. LGE is contraindicated in patients with implanted devices, which are non-MRI safe or cause significant imaging artifacts (5). Furthermore, cases including co-morbid respiratory issues limiting the length of breath-hold and claustrophobia are not ideal for LGE MRI. There is a clinical need for alternative, reliable imaging methods for the detection of cardiac scar.

Coronary computed tomography angiography (CTA) is an alternative. There are a large number of situations where CTA is the first-line investigation (6) and is part of various existing patient pathways. It is more widely available and is cheaper than MRI. CTA can be used to screen for invasive angiography (7) and is recommended as the first-line test for coronary artery disease (8). There would be a clear clinical advantage in identifying ischemic scar in CTA.

The accuracy of detecting scar visually on routine coronary CTA is unknown but, as we demonstrate in this study, it is likely related to operator experience and can be inconsistent. Iodinated contrast agent enhanced CTA have been shown, in a limited case series, to detect scar with comparable accuracy to LGE MRI (9) and electro-anatomic mapping (10); however, these earlier studies have proven hard to reproduce and are not widely used. Dual-source Computed Tomography scanners, with increased soft tissue discrimination, are a possible method of scar detection from CTA image intensities alone (11) but due to high costs and recency of their development, they are not commonly available. Due to their lack of widespread availability, there are no widely accepted clinical protocols for determining scar from dual-energy CTA. Akinetic regions are good indicators for scar (12); however, CTA motion imaging requires a high radiation dose. Ideally, scar would be rapidly and automatically detected from a standard single CTA image. This would allow the likelihood of scar to be provided on-site, at scan time to triage cases, improve scan read times, and guide clinical decision-making.

Anatomy-based prediction from a single acquisition is an alternative method of scar detection. Well-established methods of automated segmentation of CTA (13) make it possible to extract anatomical features from routine single frame scans. Left ventricle (LV) shape extracted from MRI can be used to identify scar across the whole ventricle (14). Localized LV wall thinning has also been shown to be indicative of infarction on non-gated CTA scans (15). Consistent with MRI (16), thinning in CTA has been retrospectively shown to align with scar locations confirmed by invasive electro-anatomic mapping (17). However, current wall thickness based scar detection uses a wall thickness threshold (18), which will cause miss-classification of scar due to variability in heart size. Previously proposed biomarkers for detecting scar are based on single variables, need specialized imaging, or require user-defined thresholds. We aim to perform scar detection in CTA in a single frame, without any additional imaging or user-defined input variables.

The aim of this study was to develop an automated method of LV scar detection for CTA using deep convolutional neural networks (CNNs). Our hypothesis was that the lack of labeled training data for CT can be overcome by utilizing a common data format between CTA and MRI to allow use of clinical gold standard LGE data for training. Such a method could increase both accuracy and reading time in CTA analysis.

## 2. Materials and Methods

### 2.1. Data Sources

The data were primarily retrospective data collected from previous studies, which were approved by a regional Research Ethics Committee (reference ID 264642) and conformed to the Declaration of Helsinki. A portion of the data for both MRI and CTA datasets were collected as part of a prospective study (reference ID 15/LO/1803) for which all participants provided written, informed consent.

Additional anonymized, retrospective cases for algorithm validation were provided as part of a data sharing agreement between Sheffield Teaching Hospitals and Kings College London (KCL).

### 2.2. CTA Test Dataset

Twenty-five CTA datasets were used with their meta-data displayed in Table 1 and clinical reason for the scans in Table 2. The mean age was 65, and 72% were male. Patients were retrospectively identified who had an MRI with LGE followed by a CTA scan, allowing us to estimate a scar ground truth in CTA using data from MRI. The MRIs were all within 2 years prior to the CTA (mean difference of 122 days). Potential effect on the ground truth quality from the lag between scans is discussed in the Supplementary Material. These MRIs are used to generate ground truth for testing purposes only and are not seen by the network or clinicians.

**Table 1:** Demographic information for both magnetic resonance imaging (MRI) training dataset and the computed tomography angiography (CTA) testing datasets.

	**MRI Dataset**	**CTA Dataset**
**Gender**		
Male	136	18
Female	52	7
Unknown	12	
Scar	83	10
No Scar	117	15
**Age groups**		
<60		5
60 - 64		7
65 - 70		8
>70		5

**Table 2:** Breakdown of indication for imaging in both magnetic resonance imaging (MRI) and computed tomography angiography (CTA) datasets.

**MRI Dataset**	
Chest pain clinic attendees with no infarct or evidence of cardiovascular disease (CVD)	31
Healthy volunteers from previous studies	50
Enrolled in previous studies at KCL with scar on LGE	8
Patients receiving pacing devices or defibrillators due to heart failure	111
**CTA Dataset**	
Patients who attended a chest pain clinic	11
CRT implant candidates	3
Ventricular tachycardia ablation treatment planning	5
Decreased LV function	3
Aortic valve replacement planning	2
Ischemic heart disease	1

The CTAs were all standard first-pass single-phase images with no additional iodinated contrast agent or ionizing radiation dose beyond normal clinical practice for the site. Single bolus of contrast (at 5 or 6 mL/s dependent on patient size) with saline flush was used in both sites. Three of the CTA cases had artifacts due to existing pacemaker leads and other implants. The scanner models used are discussed in Supplementary Table 3).

**Figure 1:**
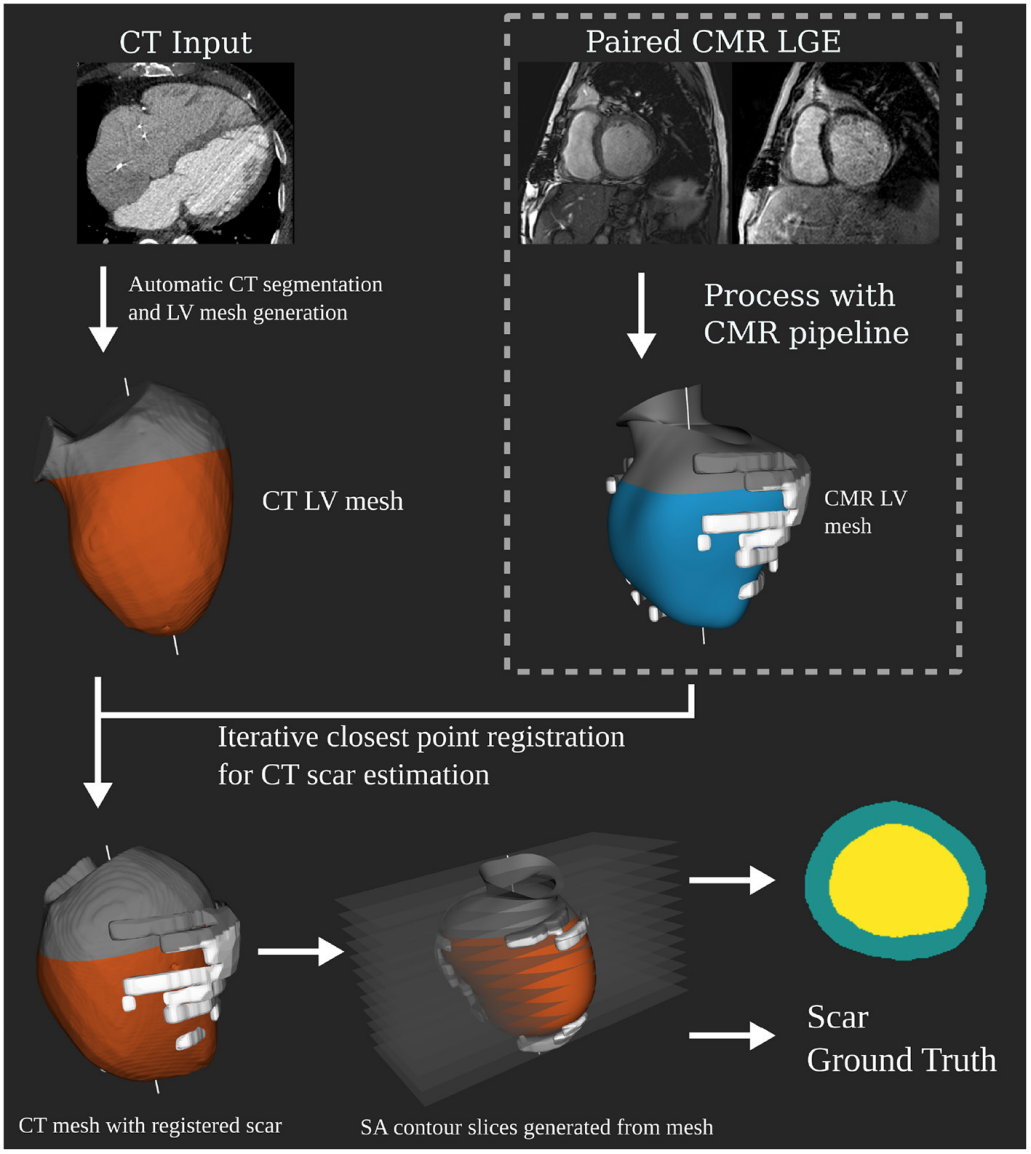
Independent computed tomography angiography (CTA) test dataset generation pipeline. Showing how the CTA anatomical masks are created as input for the network at test time. Registration of the 3D scar segmentation from paired late gadolinium enhancement (LGE) magnetic resonance imaging (MRI) is performed to determine scar ground truth for the CTA anatomical mask slices.

CTA datasets were segmented automatically using a tool provided by Siemens Healthineers as prototype software, which was previously described in Behar et al. (19). This generates 3D surface meshes of the LV without any operator input. LGE MRIs were segmented using the same tool but with manual input for both shape correction and scar identification. This produces a 3D surface mesh of the LV and scar segmentations to the same scale as the CTA segmentation, resolving any difference in resolution between the two modalities. Iterative closest point registration was used to produce a registration translation between the MRI and CT anatomical meshes using their long axes and valve intersection points. This translation was then applied to the MRI scar mesh to obtain a CTA scar mesh estimate (Figure 1). A mean difference of ±19% of the blood volume was observed between MRI and CTA meshes of the endocardium. Differences in image acquisition and the amount of aorta included in mesh segmentations between modalities account for these differences. Scans being acquired at different time points would also contribute to variations. Anatomical slices were then generated using the same slicing procedure described for the MRI dataset. This produced 420 test slices, 133 with scar present. These slices are in the form of segmentation masks, which are the input to the network.

### 2.3. Clinician Manual CTA Dataset Classification

Three independent clinical observers examined the CT dataset separately. These were either level II or III Cardiac CT practitioners with between 3 and 10 years experience each. For each CT case, they were requested to detect, as a binary classification, scar presence in the apex, mid, and basal regions. These were provided with the full CTA image stack. This can be compared directly to the task the automated system will perform, which is accurate to the slice by working off the CT image stack only.

They were also asked whether their clinical decision-making would be influenced by the presence or absence of myocardial scar in each case. Also whether an automated scar prediction tool would improve scan reading time.

### 2.4. MRI Only Training Dataset

Note that 200 MRI cases, independent of the CTA cases, were used for the training dataset. Short axis (SA), 4 chamber cine, and matching LGE images were used. Ages for MRI were not available due to automated anonymization steps prior to us receiving the data. Gender information was missing for 14 patients. Gender and scar presence information are presented in Table 1. Clinical indications for the scans are shown in Table 2. MRIs were acquired using a mixture of different scanner models and magnetic strengths as they came from different sites. Note that 1.5T scans accounted for 60% of the dataset with the remainder being done with 3T models. As described below, segmentation and 3D model creation with normalized scaling is done prior to the data being seen by the network, so this does not affect prediction.

**Figure 2:**
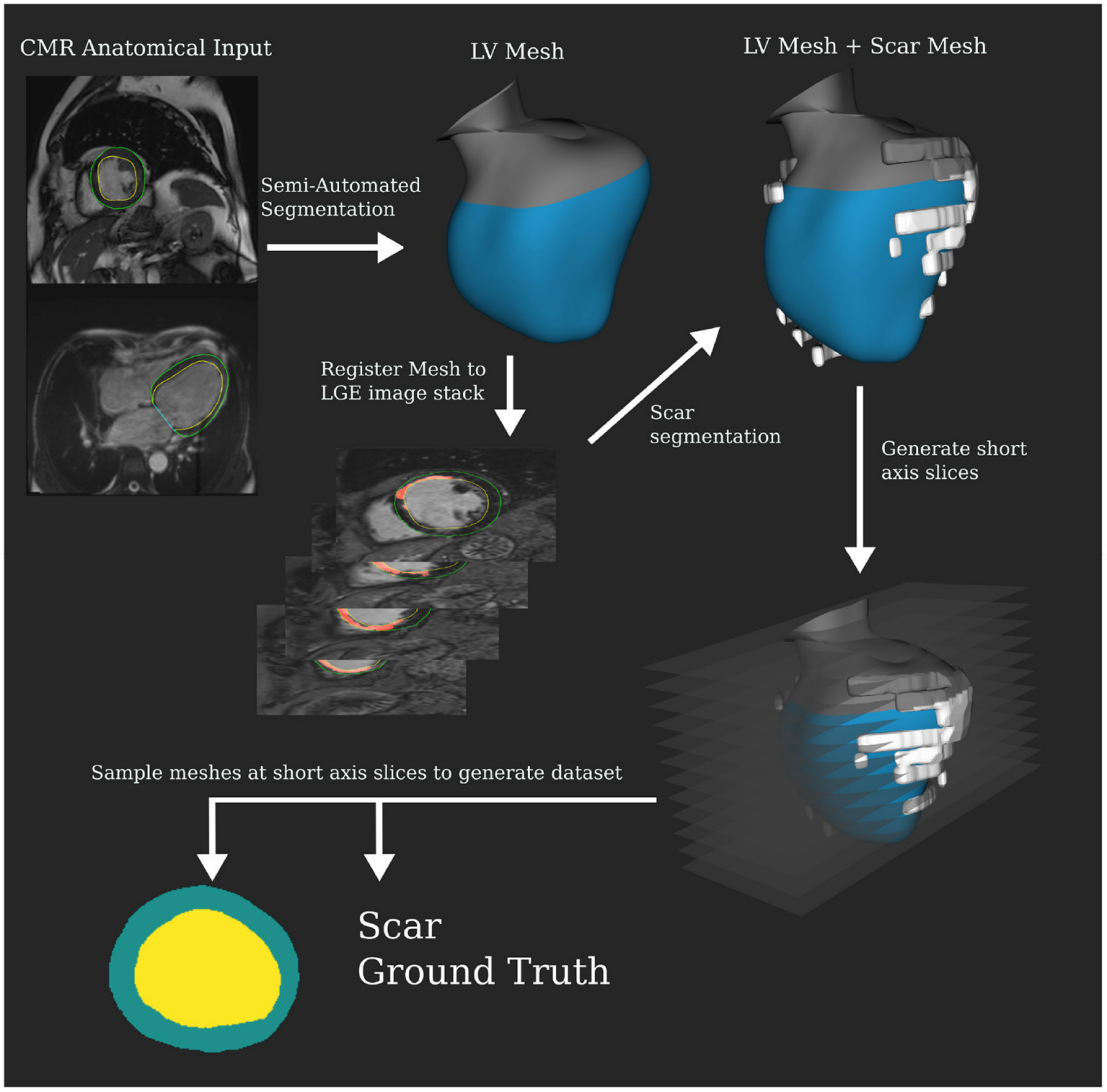
Magnetic resonance imaging (MRI) dataset processing pipeline for training and validation.

Semi-automatic segmentation was carried out on the cine MRI to produce an LV mesh, which was then registered to the LGE images to perform the scar segmentation. The segmentation tool was the same as for the CT but manual correction was required to get a good result for the MRI data. Scar was segmented manually from LGE. Custom slicing and processing code were developed using the VTK C++ library (20) to randomly sample slices from the 3D mesh segmentations. The long and short axes for the meshes were calculated via principal components analysis. Twenty-five slices were made along the short axis at regular intervals starting from 20% of the total length above the apex until the aortic valve. Intersecting contours were converted into 256 × 256 pixel anatomical masks for myocardial wall. Scar mesh intersection was calculated to determine the slices' binary ground truth. Slices were excluded if they were self-intersecting, from hitting valves, or under 50 pixels in total volume. Note that 2806 valid slices were extracted for the MRI dataset, 1006 with scar present. In the Supplementary Material, we discuss distributions in the dataset driving slicing protocol design. Figure 2 shows this pipeline that produces segmentation masks, which are used as the input to the network.

### 2.5. Classification Network

The CNN and a testing framework were implemented in Python 3 with CNNs implemented and trained using the PyTorch library version 0.4.1 (21). A network based on VGG16 (22) was used as the common CNN. In the Supplementary Material, we discuss network design considerations and alternative topologies that were explored.

The input to the network is the segmentation mask slices, which are a common format produced by either the MRI or CTA processing pipelines. This means the network is not aware of the imaging modality used to produce the data it is seeing. Input masks were converted to polar coordinates from the center of blood pool mass. This was found to increase performance in early experiments as it removes location as a variable in the data. The input images were 256 × 256 pixels with a resolution of 1 mm and 360/256◦ in the radial and circumferential coordinates, respectively. Images were padded at the base with zero-valued pixels, compensating for cases where the center of mass was near an edge, to bring them back to 256 × 256 pixels. This format provides the network with a mask showing the change in thickness around the slice.

#### 2.5.1. MRI Training and Validation

Stochastic gradient descent was used to optimize the networks. Focal loss (23), a modified version of cross-entropy loss, designed to compensate for class imbalances and easy vs. hard classifications was used. γ acts as a tunable focusing parameter. An additional tunable parameter α is added as a weighting to address class imbalances. Equation 1 shows the loss function where *p*_*t*_ is probability of correctly classifying whether an image's class is *y* = 1.

(1) $$\begin{array}{l} FL\left(p_{t}\right)=-\alpha {\left(1-p_{t}\right)}^{\gamma }log\left(p_{t}\right) \end{array}$$

Ten-fold, patient-wise, cross-validation with class balancing was used. The validation statistics were calculated using the results of test sets in all folds. Networks were trained for 100 epochs per fold, which was found to be sufficient (see Supplementary Material). The hyperparameters were tuned using the particle swarm algorithm described in the Supplementary Material.

#### 2.5.2. CTA Testing

**Figure 3:**
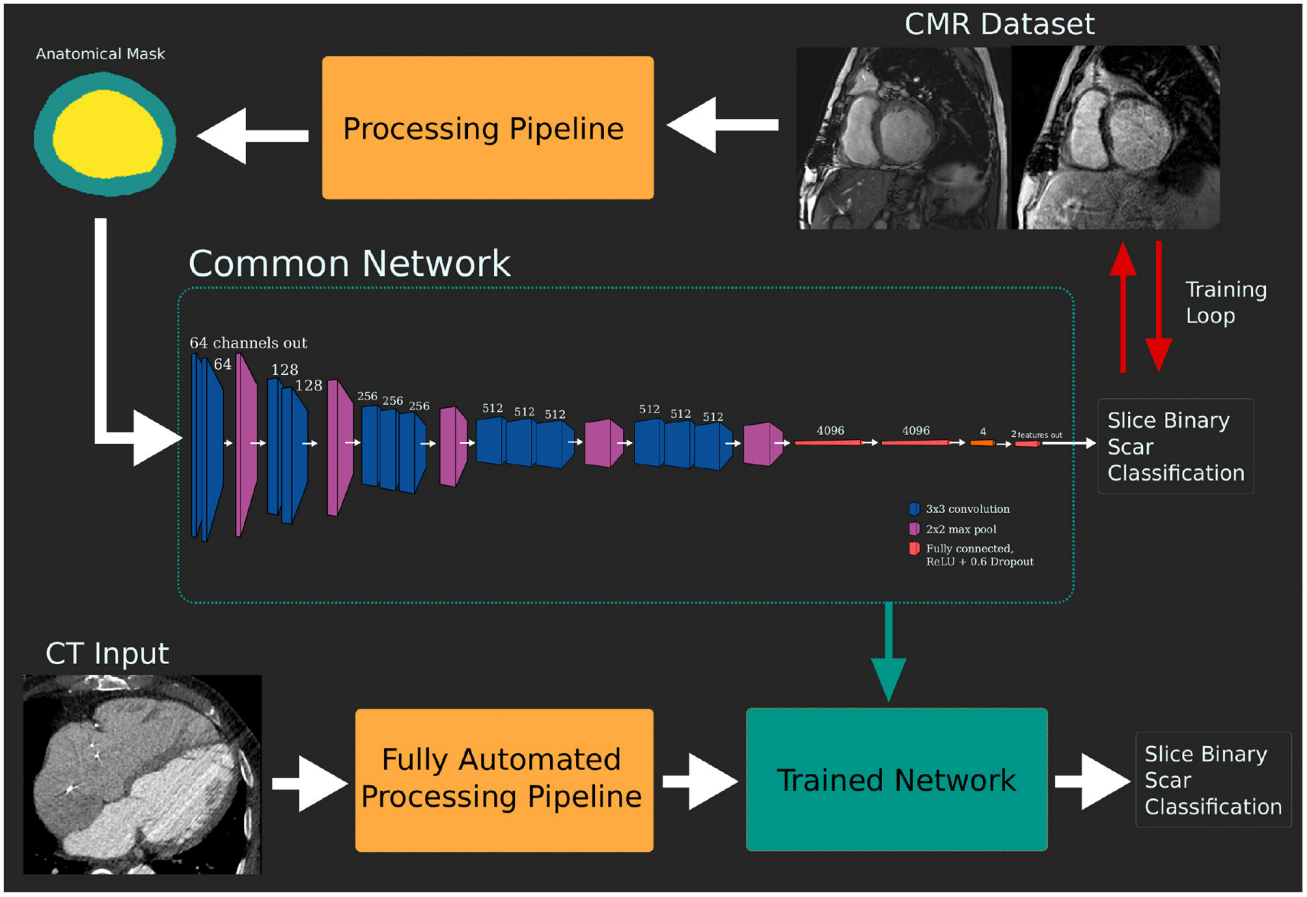
Pipeline of model training and testing. The network is trained using the dataset derived from the gold standard late gadolinium enhancement (LGE) magnetic resonance imaging (MRI) dataset. This trained network can then be tested against the target modality dataset without additional retraining.

After training and validation of the common CNNs using the MRI dataset, the network was tested against the independent CTA dataset to demonstrate the imaging modality independence of the network. Using the complete MRI training dataset, without seeing any of the CT test cases, the network was re-trained for 500 epochs. The network did not see any data, CT or MRI, from the test dataset during training. Figure 3 shows the pipeline of training and subsequent testing with the two-branch VGG variant as an example.

## 3. Results

### 3.1. Optimization and Cross-Validation Results With MRI Dataset

Accuracy was calculated using predictions of all slices when they were in the test set, which happens for each slice once in the cross-validation. Folds were generated ensuring all slices from each patient were in the same fold and that the folds contained a balanced proportion of scar slices.

(2) $$\begin{array}{l} \text{Accuracy}=\frac{\text{Sum of correct predictions from test set for all folds}}{\text{total number of samples}} \end{array}$$

Accuracy on the MRI dataset was 84.7% slice accuracy (AUC: 0.896, sensitivity: 0.76, specificity: 0.89, 95% CI: [0.885–0.906]). Table 3 displays the optimum hyperparameters tuned with the particle swarm algorithm.

**Table 3:** Results of optimizing network and network hyperparameters using the particle swarm algorithm (discussed in the supplement).

	**LR**	**M**	**Batch Size**	**γ**	**α**	**Accuracy**	**AUC**
**VGG**	0.009	0.73	10	1.560	0.6	84.7%	0.896
**CT Test**						88.3%	0.901

*Computed tomography angiography (CTA) test performance with the common network having been retrained only with the entire magnetic resonance imaging (MRI) dataset for 500 epochs. LR, learning rate; M, momentum; AUC, Area under receiver operator characteristic curve*.

### 3.2. Clinician Reading of CTA Dataset

The clinicians had, in order of least experience to most, accuracy of 60% (sensitivity: 0.69, specificity: 0.46), 71% (sensitivity: 0.53, specificity: 0.54) and 67% (sensitivity: 0.84, specificity: 0.46) compared against the LGE-derived ground truth.

It was reported that clinical judgment would be assisted by an automated suggestion of scar location in 20 cases by two or more clinicians. Two or more also reported 18 cases they said the automatic system would have sped up their assessment. There were only four cases they all agreed they would not be helped by an automated tool.

Fleiss' kappa was calculated, in order to assess the inter-operator agreement between the clinicians, at κ = 0.49, which is classed as moderate agreement.

### 3.3. CNN Testing With CTA Database

**Figure 4:**
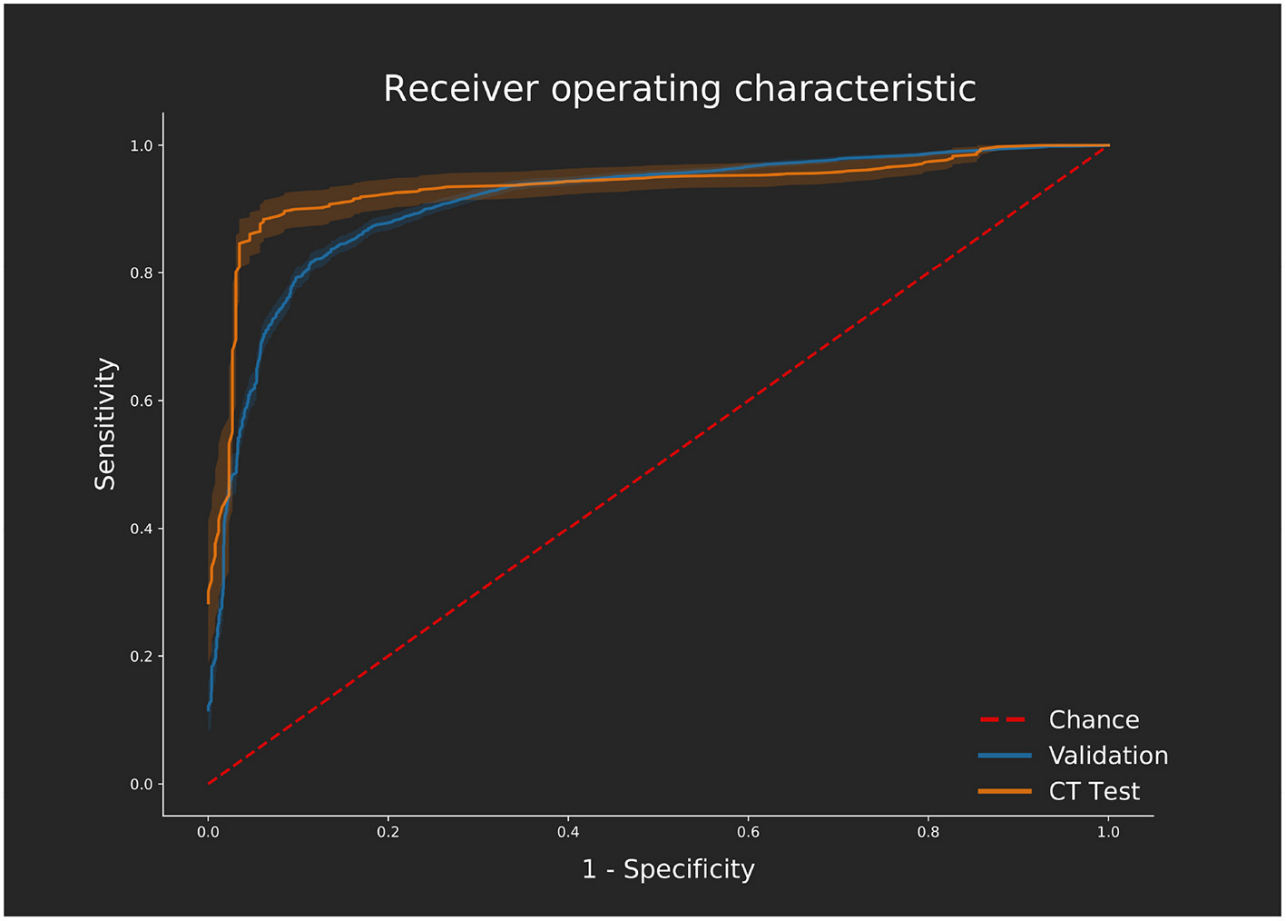
ROC curves displaying the performance on the validation [magnetic resonance imaging (MRI) dataset] calculated across all 10 folds of cross validation using the optimum hyperparameter tuning. Performance of the independent computed tomography angiography (CTA) test set using the same network, trained with the MRI dataset only, is also shown. ROC curves were generated using the Scikit-learn (28) implementation varying the threshold required for a positive classification. Confidence is shown with 1 standard deviation from the mean sensitivity and specificity values shown for each curve as calculated using bootstrapping with replacement.

The network was trained using the optimum hyperparameters using the entire MRI dataset for 500 epochs. Prediction accuracy on the CTA dataset was 88.3% (AUC: 0.901, sensitivity: 0.85, specificity: 0.90, CI: [0.867–0.934]). There was no difference found in performance between the KCL and Sheffield cases since sources of variation such as operator and scanner model are handled by the automated segmentation tool. Similarly, the artifact cases did not have diminished performance. The network processes the entire CTA dataset in 5.85 s. Including fully automated segmentation and data processing, the time taken for one CT case to be predicted using this method would be 1 min total on the hardware we tested on, varying only on the size of CTA to segment. Figure 4 shows receiver operator characteristic curves comparing the network performance on both the CTA datasets and the MRI set during cross validation.

For comparison to the clinician manual reading, two cases were not identified as having scar by all clinicians but were correctly picked up by the classification network. The network outperformed the clinicians on both sensitivity and specificity. Improved detection rates of ischemic scar in CTA would be possible by combining the network output with clinical opinion, as well as increased reading speed.

**Figure 5:**
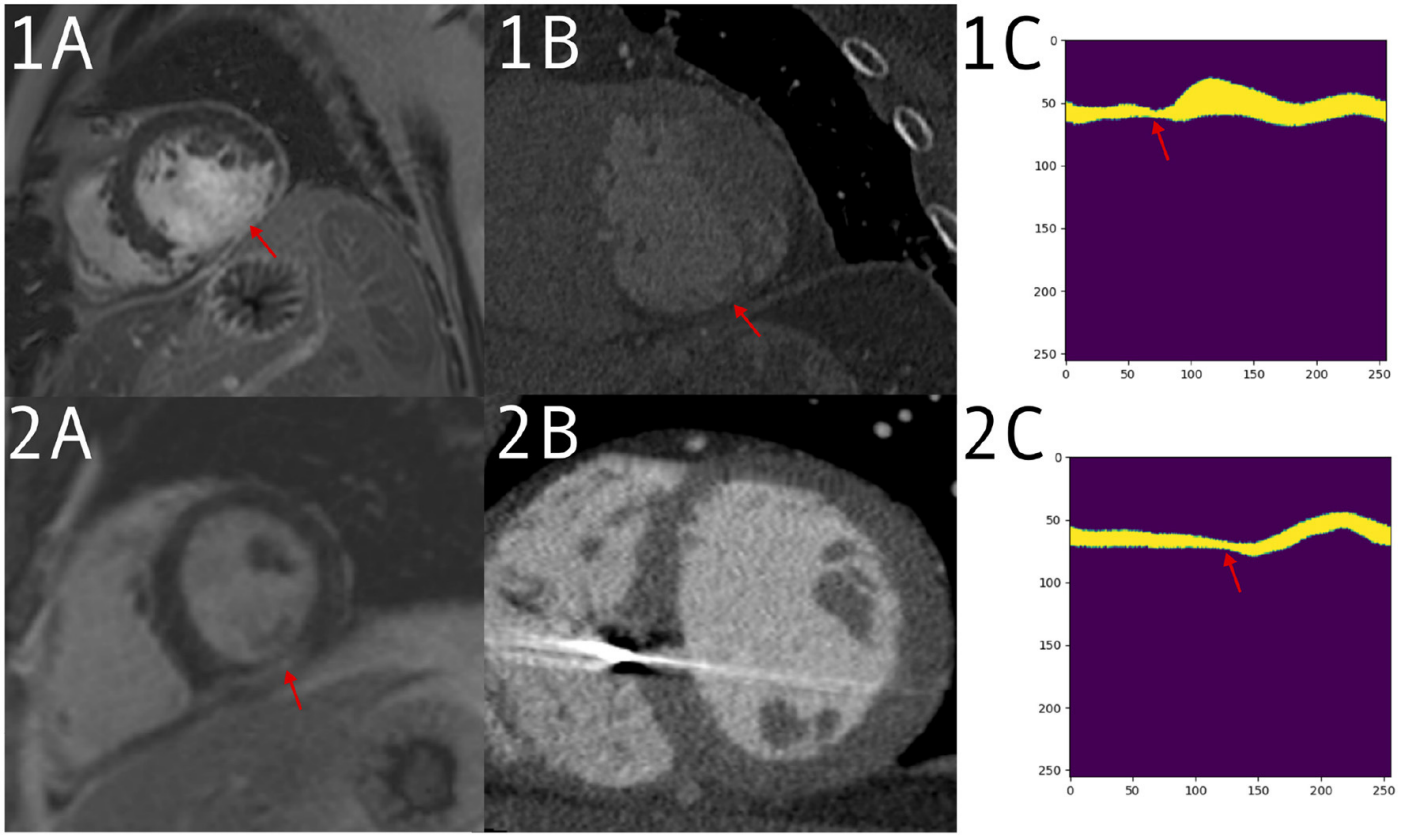
**1:** Patient with a large defect, detected correctly by both clinician and prediction network. Imaging acquired for valve replacement procedure and decreased left ventricle (LV) function from suspected infarction. **(1A)** A large defect on the late gadolinium enhancement (LGE) scan. **(1B)** The computed tomography angiography (CTA) with thinning visible in the same region. **(1C)** The input to the network for a mid-ventricle slice, in the form of a polar coordinates myocardium mask derived from the CTA segmentation. Thinning can be seen around the red arrow. **(2)** Patient with a smaller defect and less remodeling, which was detected by the network but not the clinician. Imaging performed for VT ablation planning. **(2A)** A positive LGE area on a mid short-axis slice. **(2B)** A CTA slice without an obvious defect visible. **(2C)** The polar mask with a change in anatomy, which is picked up by prediction network as a possible scar.

Figure 5 shows examples from the CTA dataset, one which was correctly identified by the network and the clinician and one which was only detected by the network.

## 4. Discussion

This study shows that ischemic scar presence can be detected in an LV SA slice using only anatomical information from a single acquisition in CTA. The experiments using MRI-derived data shows this method can be used to detect scar presence in a SA slice without the need for LGE enhancement. After training a CNN, using the clinical gold standard of MRI with LGE as ground truth, it can detect scar using data derived from CTA in a fully automatic manner. This allows for automatic scar presence detection and apical to basal location using only a CTA scan, without the need for any additional scar-specific imaging. The cardiologist manual reading experiment showed this task is difficult and inconsistent between readers. The automated CTA detection compared favorably to manual reading of the images. Since it is completely automatic for CTA, our method could be integrated into existing pipelines to increase the accuracy and speed of clinician CTA reading.

Testing the CTA dataset showed comparable results to the cross-validated accuracy results for the MRI dataset. A challenge in this work was obtaining the CTA database. To generate the ground truth as described, both a CTA exam and a recent MRI with LGE were required. It was not common for cardiac patients to have both types of imaging in the two sites from which we had data available. A larger test dataset would be desirable but the similarity between the CT and MRI dataset results indicates the method works independently of imaging modality due to the segmentation mask based input to the CNN.

In CTA images, additional information about scar presence may be available in the gray values; however, a large database of paired CTA and MRI data would be required for each training case to estimate ground truth, making such an approach impractical. Even using approaches such as transfer learning a much larger dataset would need to be compiled. We explore other possible input formats other than single slice segmentation masks, including regional 3D meshes and multi-slice approaches, in the Supplementary Material.

Our results compare favorably with previous methods using anatomy to measure scar presence. Higher accuracy has been described predicting scar based on anatomical shape models, but only on a whole ventricle level and requiring two frames of MRI data that would require Supplementary Figures to work with CTA (14). Our CTA results are similar to previously reported AHA level scar classification in CTA using biomechanical modeling to estimate myocardial strain, but these have only been shown in a small sample of canine hearts (24). Voxel methods with high accuracy have been reported using no contrast motion based methods; however, all such methods are in MRI and require multiple frames (12, 25). For these to work with CTA, non-routine sequences with higher radiation doses would be required. For this reason, we argue our method would provide benefit in supplementing routine CTA imaging and could therefore be easily integrated into an existing workflow. Contrast-free scar detection in echo has been shown in small pilot studies but would be highly operator dependent compared to our method, which is fully automated for CT (26). Our method can be added onto any automated segmentation method where a valid mesh is produced; it is not tied to the automated tool we have used for this study.

Some proportion of variance in anatomical remodeling with scar would not be detectable in the data format supplied to the model, either due to atypical presentation or only subtle remodeling. Additionally, the age of scar was not known in our dataset. Some of the scars in our dataset may have been recently formed and would have minimal remodeling (27). Additional patient information such as time since a cardiac event may improve the prediction. In the Supplementary Material, we show our method outperforms direct measurements of wall thickness, demonstrating the strength of this method compared to simpler approaches and indicating thinning alone is not enough to indicate scar with this accuracy. We also show there was no performance loss on varying heart sizes, which was demonstrated with the distributions of myocardium thickness in incorrectly classified slices.

It is possible to apply this method automatically on CTA scans, providing clinicians with likely scar slice locations without any additional input required. Such a method could be added automatically to CTA scan reports, providing additional information to help screen for LGE MRI, aid in diagnosis, and decrease reading times.

### 4.1. Limitations

This approach is designed for ischemic scar detection. In the Supplementary Material, we show examples of the method performing poorly on non-ischemic scar from hypertrophic cardiomyopathy (HCM) where anatomical biomarkers are different. Additional datasets may make it possible to predict non-ischemic scar; however, either automated exclusion by detecting HCM as a pre-prediction step or utilizing clinician judgment to avoid known atypical anatomy would avoid this limitation. Further datasets containing non-ischemic scar could increase the generalizability of our method to detecting non-ischemic conditions.

Scar presence was treated as a binary classification per SA slice, without a prediction of transmurality or percentage myocardium affected. Scar burden is challenging to estimate with a single phase of anatomy. With a larger CT-MRI paired dataset, other CNN-based methods may be able to give estimates of scar burden or more detailed localization, including transmurality. This is not possible with inputs based from single frame segmentations. This limits the usefulness of the method presented here as a stand-alone prognostic tool; therefore, we present it as useful as an automatic indicator to increase reading speed and accuracy for skilled clinicians or to indicate the need for follow-up imaging.

Observable remodeling will differ with the age of the scar as well as the size. Without knowing the age of the scar, which may not be available in many clinical settings, it would not be possible to control for this variable. This would limit the clinical utility in determining tissue viability using this method alone; however, in such cases, scar-specific imaging would be recommended. Applications such as pacemaker lead placement and improving manual reading speed would still benefit from a binary classification. Triaging scans as part of an existing patient pathway would also be possible with this method.

The principal aim of the study was to develop a method of scar detection on routine CTA images. An impact assessment of the utilization of this technique was not performed in terms of clinical outcomes or reporting times; however, the clinicians involved reported their reading would have been faster with indications of regions of potential scar from an automated tool. Ongoing work, therefore, aims to investigate this method in a clinical setting as well as improve the output based on clinical utility.

### 4.2. Conclusion

Ischemic scar presence in the left ventricle short-axis slices can be detected using a CNN and anatomical information extracted from a single acquisition of CTA. We demonstrated our method performing well in both MRI and CTA. For CTA, there is no automated scar detection method and clinician detection will vary based on operator experience. This method outperformed manual reading, requires only routine CTA scans, and is automatic. The proposed method provides a near zero cost automated scar presence detection with apical-basal location. Applying this approach to routine cardiac CTA images can be used to automate screening for potential infarcts, to triage images, and to improve scan reading times.

## Data Availability Statement

The data analyzed in this study is subject to the following licenses/restrictions: The data used for this study is imaging data from 2 NHS hospitals for which ethics was acquired for specific use in this study. Requests to access these datasets should be directed to hugh.o'brien@kcl.ac.uk.

## Ethics Statement

The studies involving human participants were reviewed and approved by UK Health Departments' Research Ethics Service. The patients/participants provided their written informed consent to participate in this study.

## Author Contributions

HO'B, TK, RR, KR, PM, and SN: conception and design. HO'B, JW, BS, JG, MO'N, RR, KG, CR, and JT: analysis and interpretation of data. All authors were involved in drafting of the manuscript or revising it for content. All authors approved the final manuscript submitted.

## Conflict of Interest

The authors declare that the research was conducted in the absence of any commercial or financial relationships that could be construed as a potential conflict of interest.
