# Supplementary material for: Automated Left Ventricle Ischemic Scar Detection in CT Using Deep Neural Networks
Source: Front Cardiovasc Med. 2021 Jul 2;8:655252. doi: 10.3389/fcvm.2021.655252 (PMC8283258; doi:10.3389/fcvm.2021.655252)
Supplement: Supplementary file 1 [file Data_Sheet_1.DOCX]

**Supplemental Experiments**

Additional experiments were carried out with the goals of evaluating the databases and exploring alternative methods of classification.

**Contents**

[1.1 Analysis of MRI Training Database 3](#_Toc17247)

[1.2 Average Wall Thickness Based Classifier 4](#_Toc17248)

[1.2.1 Results 7](#_Toc17249)

[1.3 Number of Epochs Required Analysis 7](#_Toc17250)

[1.4 Network Design 8](#_Toc17251)

[1.4.1 Particle Swarm Optimisation 8](#_Toc17252)

[1.4.2 Results and Discussion 9](#_Toc17253)

[1.5 Does 3D MRI Improve Classification? 10](#_Toc17254)

[1.5.1 Multi-slice SA 10](#_Toc17255)

[1.5.2 Surface Mesh predictive network 11](#_Toc17256)

[1.5.3 Discussion of 3D Methods 12](#_Toc17257)

[1.6 Downsampling MRI Input Data 12](#_Toc17258)

[1.7 HCM Influence on CT Testing Dataset 13](#_Toc17259)

# 1.1 Analysis of MRI Training Database

Figure S1 shows distributions of valid slice locations (not self-intersecting, intersecting valves, or under 50 pixels in total volume), scar locations and incorrect scar slice predictions in the apical-basal direction. We can see there was a near uniform distribution in valid slice locations. A small increase at the most basal locations was seen due to differences in valves location, meaning the number of slices was inconsistent between cases. Mean number of scar slices per location was 143 (max: 157, min: 126), with a small increase in slices towards the basal to mid cavity. With this apical-basal distribution, we would expect no differences in performance accuracy depending on slice location.

Across the slices, apical and basal slices were the worst performing (Figure S1). For the apical, case we attribute this to the volume of tissue available and therefore the amount of information available to the network. Inaccuracies in mesh formation may also be to blame causing the appearance of thinner tissue towards the apex. For these reasons, the apex was a source mostly of false positives. For the basal slices, the opposite is true where changes in anatomy would be less visible due to the thicker wall volumes towards the base. A proportion of false positives were also found here as the mesh curved to join with the aortic valve, creating the appearance of anatomical change in a 2D slice. Partial volume effects near the apex make it difficult to segment scar presence using LGE MRI [1]. Similar effects at basal slices near the aortic outflow tract can cause false positive LGE MRI segmentations. This shows that the method is most accurate in the locations where we were most likely to find scar and the anatomy would be expected to have a mostly constant shape in control cases.

The distribution of misclassified slices lead to the decision to automatically exclude the most basal and apical slices during processing. 20% above the apex and 10% below the base were automatically excluded which ensures the network is trained and tested on the portions of anatomy where scar biomarkers are most identifiable. This meant that there was more consistency between cases in terms of which slices were included, defining a region of the LV we expect our method to perform well on.

No bias was found in median thickness of the myocardium affecting how easy it was to classify a slice (Figure S2). The distribution of incorrect slice thicknesses follows closely to the thickness distribution in the CMR dataset as a whole. Beyond removing the most apical and basal slices, as already discussed, no weakness was found limiting the usability of the method depending on heart size or median wall thickness.

Apical-Basal CMR Slice Distribution

0.2

0.3

0.4

0.5

0.6

0.7

0.8

0.9

Relative Aplical-Basal Position

0

100

200

300

400

Slices

Apex

Base

Apical-Basal Scar Slice Distribution

0.2

0.3

0.4

0.5

0.6

0.7

0.8

0.9

Relative Aplical-Basal Position

0

50

100

150

Slices

Apex

Base

Incorrect Slice Prediction Distribution

0.2

0.3

0.4

0.5

0.6

0.7

0.8

0.9

Relative Aplical-Basal Position

0

10

20

30

40

Slices

Apex

Base

Figure S1: Top: Relative distribution in the apical-basal direction of slices in the whole MRI dataset. Middle: MRI slices with positive scar on LGE. Bottom: distribution of MRI database slices incorrectly predicted by all networks after optimisation. 0 indicates the apex and 1 the base.

Slice Median Thickness Distribution

4

5

6

7

8

9

10

11

12

Median Slice Thickness (cm)

0

200

400

600

800

Slices

Incorrect Slice Median Thickness Distribution

4

5

6

7

8

9

10

11

12

Median Slice Thickness (cm)

0

10

20

30

40

50

Slices

Figure S2: Top: Relative median myocardium thickness for slices in the MRI dataset. Bottom: Median myocardium thickness in slices which were incorrectly predicted in the 10-fold cross validation 0 indicates the apex and 1 the base.

# 1.2 Average Wall Thickness Based Classifier

Changes in wall thickness are associated with scar [2]. There has been some success in using thickness thresholds to identify scar for cardiac ablation procedures [3]. Our MRI dataset was utilised to create a classifier for scar thickness rather than picking a fixed value. We aimed to produce a thickness classifier using individual measurements and mean thickness values.

From the centre of mass in the blood pool, measurements were taken at regular intervals of 20^◦^. Average thickness values were compared between scar and non-scar patients to ascertain whether there was a significant difference, as defined by a p-value of under 0.5 from a t-test, between scar and no-scar patients. Mean thickness measurements were also compared between slices with and without scar regardless of whether the patient had some scar.

A support vector machine (SVM) implemented using scikit-learn [4] was then trained on the measurement data to establish whether measurements of local thickness could be used to detect scar presence. Other common classification techniques including random forest and linear regression were tested but SVM was the best performing method. Two variations of inputs were tested:

- **Individual measurements**, where each line from the blood pool to the epicardium edge was considered a data point. The measurement intersecting with scar on LGE tissue was used as ground truth.
- **Mean slice thickness** and whether any scar was in the slice. This second case is more analogous to the data shown to the networks but lacks any local change data in the input.

Both variations used a 0.33:0.66 test train split with class balancing.

## 1.2.1 Results

The mean thickness of slices across the whole heart for patients in the scar (4.13 mm) and no scar groups (5.81 mm) was significantly different as tested with a t-test (t = 16.69, p *<* 0.05). The mean thickness measurements of scar slices (4.13 mm) and non-scar slices (5.65 mm) were also significantly different (t = 36.97, p *<* 0.05).

An SVM was trained on the measurement data individually with each measurement being given a binary class dependent on whether it intersected with scar. For this variation, a total number of 53314 measurements were provided with 3786 of them intersecting with scar. The accuracy obtained was 32.85 %. Looking at table S1 we can see that there is a very high rate of false positives. This shows that a method such as setting a threshold or otherwise using local thickness may find scar presence but only at the expense of greatly overestimating scar locations.

Another SVM was trained with the mean thickness values across the whole slice as input. 2806 slices with 1007 containing scar were used. The accuracy obtained was 72.14 %. This loses the local specificity of the local measurements but as we can see is more accurate. This is a large improvement compared to individual measurements. Many of the slices with scar present have thicker areas or simply have large enough hearts to raise the mean slice measurement into the normal zone. A difference in the mean is not sufficient to classify on. This result informs the decision to use wall thickness as input to a scar classifier but signals more information than mean thickness or local thickness would be necessary to achieve results close to those from mechanics-based methods.

These results also confirm that there is remodelling in some patients with scar which shows up in comparisons of thickness. On average, scar slices are thinner but local measurements of wall thickness alone do not make a good classifier for scar localisation.

|  | Scar | No Scar |
| --- | --- | --- |
| **SVM on individual thickness measurements (Acc: 32.85%)** | | |
| Totals | 1209 | 16385 |
| No Scar Prediction | 85 | 4628 |
| Scar Prediction | 1124 | 11757 |
| **SVM on mean slice wall thickness (Acc: 72.14%)** | | |
| Totals | 321 | 605 |
| No Scar Prediction | 257 | 411 |
| Scar Prediction | 64 | 194 |

Table S1: SVM performance in predicting scar presence with local myocardium thickness measurements and whole short axis slice average thickness.

# 1.3 Number of Epochs Required Analysis

To ensure manageable training times for the swarm optimisation as well as more complex networks such as the two-branch network analysis of how many epochs would be required was done using the VGG network. The loss was found to decrease rapidly before tailing off before 100 epochs in a training run of 500 epochs using the MRI dataset. Tracking loss across 10 fold cross validation showed 100 epochs was enough for every fold to reach it’s lowest loss value (Figure S3). An identical training run using 50 epochs had 83% accuracy across cross validation folds. 100 was selected as a safe number of epochs to ensure most of the training gains were being captured during optimisations using the particle swarm algorithm.

0

100

200

300

400

500

Epochs

0.00

0.05

0.10

0.15

0.20

Focal Loss

Epoch Training

500

0

200

400

600

800

1000

Epochs

0.05

0.10

0.15

0.20

0.25

Focal Loss

Training loss across 10-fold cross validation

Figure S3: Top: Tracking focal loss values across epochs for 500 epochs, showing a levelling before 100 epochs; Bottom: Focal loss values across 1000 total epochs, split into 10 cross validation folds. Levelling occurs before the end of the fold’s 100 epochs.

# 1.4 Network Design

Three networks were compared for the classification task using the MRI training and validation dataset: VGG16 [5], a two-branch variant of VGG16 with a pre-trained branch and Densenet-121 [6]. Group normalisation [7] was used between layers to allow for lower batch sizes during training larger models without losing accuracy stability, otherwise memory limits of available hardware would be a concern.

The two-branch VGG network was considered to detect if a pre-trained component, trained on the image-net database (available from the PyTorch repositories), would provide the benefit of already learned features, such as edges. The results of each network were concatenated and fed into an additional fully connected layer to produce the output binary classification. This topology is shown in Figure **3**.

## 1.4.1 Particle Swarm Optimisation

The particle swarm algorithm [8] was used to optimise the hyperparameters in all networks using the MRI dataset.

For each network 10 particles were optimised for 50 evaluation runs to find optimum values initial learning rate (range: 0.001 - 0.01), momentum (range: 0.7 - 0.9), batch size (range 5 - 16) and focal loss parameters: gamma (range: 0.5 - 2.0), alpha (range: 0.1 - 1.0).

The best results from the particle swarm hyperparameter tuning for each network were shown in Table S2 in the main paper.

## 1.4.2 Results and Discussion

After optimisation VGG was the best performing network on the MRI dataset with 84.7% slice accuracy (AUC: 89.6, sensitivity: 0.76, specificity: 0.89, 95% CI: [0.885 - 0.906]). The multi-branch VGG variant performed very similarly with 83.4% accuracy (AUC: 89.6, sensitivity: 0.75, specificity: 0.88, CI: [0.886 - 0.906]). Densenet performed worst with 80.8% accuracy (AUC: 0.807, sensitivity: 0.67, specificity: 0.81, CI: [0.792 - 0.821]). Figure S5 includes the receiver operator characteristic (ROC) curve for all networks.

Training times for each run were 13 hours for VGG, 20 hours for multibranch and 8 hours for Densenet. Since the difference in performance was small, the VGG was selected due to faster training and running time. The VGG took 5.85 seconds to process the CTA dataset, while the multibranch took 8.25 on our hardware (Nvidia Titan XP). A smaller network is more portable and would run on lower memory systems as well as being more useful for batch processing large datasets.

**M**

**LR**

**Batch**

**Size**

*α*

*γ*

**AUC**

**Accuracy**

**VGG**

0.009

0.73

10

1.560

0.6

%

84.7

0.896

**VGG**

**-**

**multibranch**

0.006

0.74

9

1.77

0.73

%

83.4

0.896

**Densenet**

0.008

0.89

6

1.142

0.437

80.8

%

0.807

**CTTest**

%

88.3

0.901

Table S2: Results of optimising network topology choice and network hyperparameters using the particle swarm algorithm (discussed in the supplement). CTA test performance with the best performing network having been retrained with the entire MRI dataset for 500 epochs. LR: learning rate; M: momentum; AUC: Area under receiver operator characteristic curve.

Both of the VGG based networks performed better than the Densenet, with only a small difference between them in performance (*<* 1*.*5%). Increasing the complexity of the network with either an additional branch or dense network design did not result in any gains in performance, implying we are capturing most of the available variance in our input format already with the VGG topology. It may be possible to decrease complexity of the network further.

# 1.5 Does 3D MRI Improve Classification?

Two experiments using the MRI dataset in 3D were considered to determine whether the use of 3D inputs improved predictive power.

## 1.5.1 Multi-slice SA

First, a 2.5D method was considered where three slices were viewed by the CNN at a time. These were encoded as channels, providing the network with anatomical information for the SA slice and its adjacent apical and basal slices. The MRI dataset was processed in the same manner as the main processing pipeline. A total of 2206 sets of 3 slices were produced with 869 having scar. 10 fold cross validation was used for training and calculating performance in the same manner as the main experiments.

A small improvement in performance was achieved over the best performing CNN from the single slice experiments. A comparable 84.13% accuracy but with an AUC of 0.907 (CI: [0.896 - 0.917]).

| Multi-sliceinputVGG | | | SurfaceMeshCNN | | |
| --- | --- | --- | --- | --- | --- |
|  | No Scar | Scar |  | No Scar | Scar |
| No Scar | 1183 | 196 | No Scar | 625 | 95 |
| Scar | 154 | 673 | Scar | 120 | 121 |

Table S3: Confusion matrix for 3-slice input VGG and surface mesh classification experiments.

## 1.5.2 Surface Mesh predictive network

Sections of the endocardial and epicardial meshes were provided as input to MeshCNN, a network which takes meshes as input and performs convolutions on edges and the four edges of their incident triangles. This has shown success in classifying objects in 3D [9]. This was an attempt to test if we were throwing away data during the slicing process which would otherwise be useful for prediction. The MRI dataset meshes were divided into the AHA 16 segment model (Figure S4). Each vertex of the epicardial segment had two edges created to join it to the two nearest endocardial vertices to create a triangular face, encoding the local thickness as well as surface changes in the mesh input data.

Using the MRI dataset 3200 segment meshes were produced. A train-test split of 7:3 was used balancing for scar class between sets. The test set contained 961 segments of which 216 had scar present. The network was trained for 2000 epochs. No augmentations were applied to the data. Focal loss was used as a custom loss function with an alpha of 0.5 and gamma of 3. Meshes were normalised to a size of 2600 edges. Pooling between convolutional layers were at resolutions of 1800, 1350 and 600 edges. These hyperparameters were selected using a simple grid search optimisation. Accuracy achieved was 77.62% with a low sensitivity of 0.56.

MeshCNN

Slice Binary

Scar

Classi

fi

cation


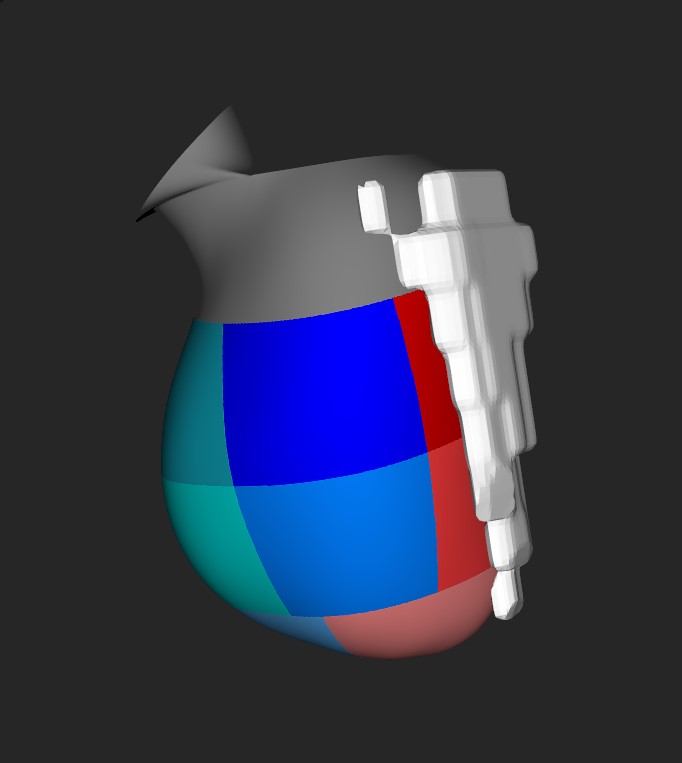


3

D Anatomical Mesh


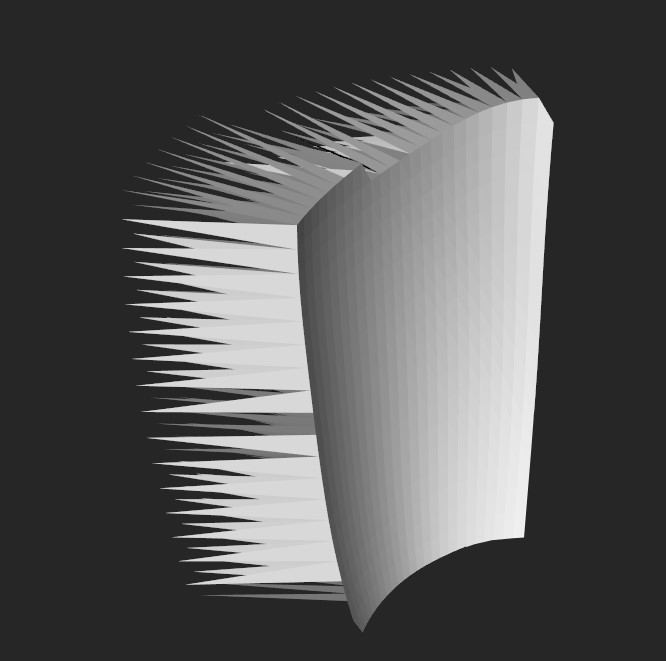


AHA segment with edges between

endo and epicardial faces

Figure S4: Method of classifying scar anatomy based on segments of the surface mesh. Endocardial surface and edges joining each vertex to epicardium shown with epicardium excluded for visibility.

## 1.5.3 Discussion of 3D Methods

The small improvement in performance from the multi-slice method over the single slice VGG method came with disadvantages. The first was a small increase in run-time due to the higher complexity of the data. The other was a loss in apical-basal resolution. The decrease in resolution also came with a small decrease in the database size, since slices at the edges could only be used once while middle slices could be used multiple times. The improvement in AUC was small and was on a dataset which minimised apical and basal slices that are more difficult to classify (Figure S1).

The surface method was not found to be viable, due both to the more limited training set and the low sensitivity.

Since it was more spatially specific without a large decrease in performance development was focused on the single slice methods.

# 1.6 Downsampling MRI Input Data

An experiment downsampling the slice masks was carried out to investigate whether enough information would be available in a lower resolution image. Since changes in thickness or shape were likely to be visible across multiple pixels this would allow us to perform the same task with small images and thus lower prediction times. The VGG network was trained using the MRI dataset with 10-fold cross validation for 100 epochs using the optimum hyperparameters found using the swarm optimisation described in the main methods. Each image was downsampled to 128x128 using nearest neighbour interpolation.

Cross validation accuracy was 81.78%, which is a 4% decrease in accuracy. The AUC was 0.87, CI: [0.859 - 0.882]. The cross-validation took 4 hours to run in total.

There is a performance hit from down-sampling which may be worthwhile in the case of lower power hardware being used. Most of the predictive power can be maintained however with a lower resolution.

# 1.7 HCM Influence on CT Testing Dataset

To determine if the method was limited to ischaemic scar three CTA cases with HCM were analysed. All three of the HCM cases performed poorly due to their atypical anatomical presentation with thickened walls but diffuse scar on LGE. Of the 45 slices from these cases, 28 were incorrectly predicted. All of these were false negatives, with the sensitivity for the CTA database decreasing from 0.85 to 0.7 if these HCM cases are included. Figure S5 shows the noticeable difference between the sensitivity with and without these cases. The AUC for the CT dataset without the HCM cases was 0.901 compared to 0.791 with them included (CI 0.749 - 0.838, sensitivity: 0.7, specificity: 0.91). In a system including this method, an appropriate design would be to have a classification system for HCM cases prior to the scar detection.

Including sufficient HCM cases in the MRI training set may also improve this result; however, due to the nature of the anatomical differences specific to HCM, this would be likely to have a negative impact on the performance on non-HCM cases.

Other conditions with atypical anatomical presentation such as dilated cardiomyopathy would be likely to behave similarly with our method. As such we would recommend this method be used in conjunction with abnormal anatomy detection and appropriate clinical judgement on this limitation.

0.0

0.2

0.4

0.6

0.8

1.0

- Specificity

1

0.0

0.2

0.4

0.6

0.8

1.0

Sensitivity

Receiver operating characteristic

Chance

VGG

VGG Multibranch

DenseNet

CT Test

CT Test With HCM

Figure S5: ROC curves displaying the performance of all network variants as in Figure **4** with additional curve for CT performance with HCM cases.

# 1.8 CT Dataset Details

Table S3 shows the models used for the CT acquisitions in the test dataset. The Siemens scanner is a dual energy scanner but since the detection method described in this work uses the segmentation of the scans as input rather than the grey values themselves this does not influence the results. No performance difference was seen between the sources of data, with 4 scar cases coming from the Toshiba scanner.

| **CT Scanner Model and Manufacturer** |  |
| --- | --- |
| Siemens SOMATOM Force | 20 |
| Toshiba | 5 |

Table S3: CT scanner models and manufacturers for the CT test dataset.

## 1.8.1 Time between MRI and CTA

The mean time between MRI LGE and CTA scans was 122 days, with the MRI always being before the CTA and within 2 years prior. Any gap between the two scans may impact the quality of the ground truth since this was acquired by registering the MRI LGE scar segmentation mesh to the CTA mesh from a different time point.

For the non-scar cases the risk of new scar was low. 10 had CTA as attendees to a chest pain clinic where there was no finding which would indicate a new scar. 3 were CTAs ordered after MRI to investigate suspected coronary artery disease after no scar shown on LGE but the patients presented with decreased LV function. One was a CRT implant case where the lag time was under a month between scans. The final was a valve replacement procedure planning scan. In this case both the network and all three clinicians agreed on no scar in all regions.

In all the scar cases there is a risk of increase scar burden due to worsening of the original condition, potentially in slices where the LGE was originally negative.

For cases under the mean time difference (N=13) there was 88.2% slice accuracy and for cases over the mean (N=12) there was 87.7% slice accuracy. This indicates there was no effect seen in our dataset but in a larger dataset this potential for error in the ground truth should be considered, especially looking at any increase in the false positive rate.

**References**

1. Turkbey Evrim B., Nacif Marcelo S., Guo Mengye, et al. Prevalence and Correlates of Myocardial Scar in a US Cohort *JAMA.* 2015;314:1945.
2. Nowosielski Martha, Schocke Michael, Mayr Agnes, et al. Comparison of wall thickening and ejection fraction by cardiovascular magnetic resonance and echocardiography in acute myocardial infarction *Journal of Cardiovascular Magnetic Resonance.* 2009.
3. Cedilnik Nicolas, Duchateau Josselin, Dubois R´emi, Ja¨ıs Pierre, Cochet Hubert, Sermesant Maxime. VT Scan: Towards an Efficient Pipeline from Computed Tomography Images to Ventricular Tachycardia Ablation in *Functional Imaging and Modelling of the Heart*:271–279Springer, Cham 2017.
4. Pedregosa Fabian, Varoquaux Gael, Gramfort Alexandre, et al. Scikitlearn: Machine Learning in Python 2012.
5. Simonyan Karen, Zisserman Andrew. Very Deep Convolutional Networks for Large-Scale Image Recognition *ICLR.* 2014.
6. Huang Gao, Liu Zhuang, Maaten Laurens, Weinberger Kilian Q.. Densely Connected Convolutional Networks 2016.
7. Wu Yuxin, He Kaiming. Group Normalization 2018.
8. Clerc Maurice, Kennedy James. The particle swarm-explosion, stability, and convergence in a multidimensional complex space *IEEE Transactions on Evolutionary Computation.* 2002;6:58–73.
9. Hanocka Rana, Hertz Amir, Fish Noa, Giryes Raja, Fleishman Shachar, Cohen-Or Daniel. MeshCNN: A Network with an Edge 2018.
